# Remodeling of the *Streptococcus agalactiae* Transcriptome in Response to Growth Temperature

**DOI:** 10.1371/journal.pone.0002785

**Published:** 2008-07-30

**Authors:** Laurent Mereghetti, Izabela Sitkiewicz, Nicole M. Green, James M. Musser

**Affiliations:** 1 Center for Molecular and Translational Human Infectious Diseases Research, The Methodist Hospital Research Institute, Houston, Texas, United States of America; 2 Université François-Rabelais, Faculté de Médecine, EA3854 “Bactéries et risque materno-foetal” and Centre Hospitalier Universitaire, Tours, France; Centre for DNA Fingerprinting and Diagnostics, India

## Abstract

**Background:**

To act as a commensal bacterium and a pathogen in humans and animals, *Streptococcus agalactiae* (group B streptococcus, GBS) must be able to monitor and adapt to different environmental conditions. Temperature variation is a one of the most commonly encountered variables.

**Methodology/Principal Findings:**

To understand the extent to which GBS modify gene expression in response to temperatures encountered in the various hosts, we conducted a whole genome transcriptome analysis of organisms grown at 30°C and 40°C. We identified extensive transcriptome remodeling at various stages of growth, especially in the stationary phase (significant transcript changes occurred for 25% of the genes). A large proportion of genes involved in metabolism was up-regulated at 30°C in stationary phase. Conversely, genes up-regulated at 40°C relative to 30°C include those encoding virulence factors such as hemolysins and extracellular secreted proteins with LPXTG motifs. Over-expression of hemolysins was linked to larger zones of hemolysis and enhanced hemolytic activity at 40°C. A key theme identified by our study was that genes involved in purine metabolism and iron acquisition were significantly up-regulated at 40°C.

**Conclusion/Significance:**

Growth of GBS *in vitro* at different temperatures resulted in extensive remodeling of the transcriptome, including genes encoding proven and putative virulence genes. The data provide extensive new leads for molecular pathogenesis research.

## Introduction

*Streptococcus agalactiae*, also known as group B streptococcus (GBS), is a common inhabitant of the human gut and asymptomatically colonizes the vagina of one-third of women [1]. However, the organism also is a major human pathogen, causing severe neonatal infections [2], and has emerged recently as a leading cause of invasive infections in the elderly [3]. GBS also has been isolated from several asymptomatic colonized and diseased animal species such as cows [4], [5]. Although GBS has no known environmental reservoir, given its widespread host, anatomic, and disease range, it is clear that the bacterium must adapt to many environmental conditions to survive and thrive.

Bacteria monitor the environment and alter gene expression in response to many factors, including temperature, pH, osmotic activity, oxygen levels, nutrient sources, and ion concentrations [6], [7]. Temperature variation is one of the most commonly encountered environmental changes, and it has been reported that bacteria can modify expression of at least 10% of their genes in response to an increase or decrease in growth temperature [8], [9], [10]. Large, dynamic changes in expression of genes implicated in metabolism, general adaptive responses, membrane structure, and virulence have been reported [8], [9], [10]. However, little is known about the influence of temperature on GBS gene expression.

To enhance our understanding of the capacity of GBS to adaptively respond to growth-temperature change, we used expression microarray analysis to evaluate global transcript changes occurring during cultivation at 30°C and 40°C. These temperatures were chosen because they correspond approximately to those occurring in patients with severe infections and high fever (∼40°C) and on the surface of the cow mammary gland (∼30°) [11], or temporary persistence in human and bovine milk [12], [13]. We discovered that GBS extensively remodels its transcriptome at various stages of growth when cultured at these two temperatures. Key themes identified by our analysis include differential expression of genes encoding hemolysins, proven and putative virulence factors, proteins involved in iron acquisition, purine/pyrimidine metabolism, and nutrient utilization.

## Results and Discussion

### Global expression microarray analysis

**Figure 1:**
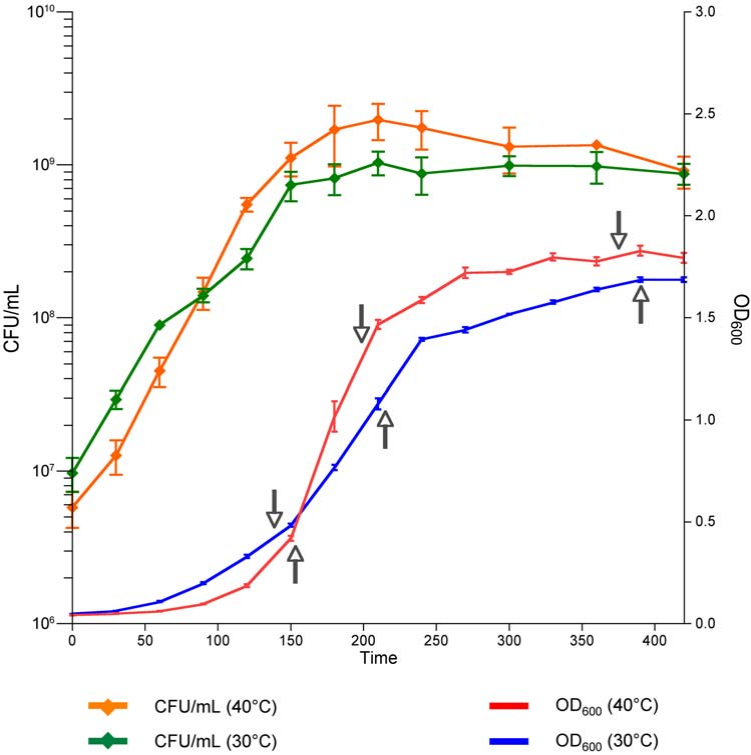
Growth curves for GBS strain NEM316 at different temperatures. Overnight THY cultures were diluted 1:50 with fresh THY broth at 30°C or 40°C and cultured in 5% CO_2_. Growth was monitored by measuring the OD_600_ and CFU counts with blood agar plates.

The time points used for global transcript analysis are shown in the bacterial growth curves presented in Fig. 1. We compared the transcript level of genes grown at 30°C and 40°C at mid-logarithmic, late logarithmic, and stationary phases. In general, we analyzed only transcripts expressed at least at one temperature for one of the three times of growth, whose expression was changed 2-fold at one temperature relative to the other temperature (i.e., 30°C and 40°C), and for which the difference in transcript level was statistically significant (*P*<0.05). Genes for which the transcript level increased or decreased greater than 1.5-fold were included when they were of specific interest and are noted as such in the manuscript.

**Figure 2:**
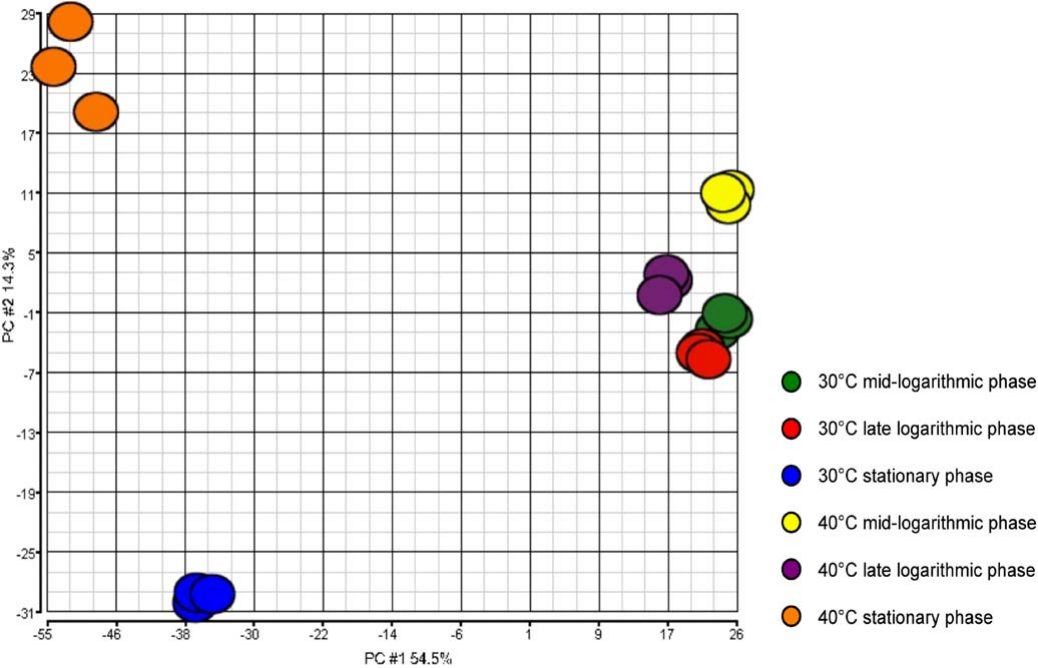
Principal component analysis (PCA) plot expression of microarray data. The figure shows transcriptome differences between strain NEM316 incubated in THY at 30°C and 40°C. The PCA plot captures the variance in a dataset in terms of principal components and displays the most significant of these on the x and y axes. The percentages of the total variation that are accounted for by the 1st and 2nd principal components are shown on the x- and y-axes labels. The results indicate that the transcriptome data are of high quality, as the triplicate samples cluster together according to both temperature of incubation and growth phase.

We first assessed chip-to-chip data variability and quality using a principal component analysis (PCA) (Fig. 2). The PCA analysis discriminated very well between the transcript data from the chips representing mid-logarithmic, late logarithmic, and stationary growth phases, at 30°C and 40°C (Fig. 2). These results indicated that the transcriptome profile data from triplicate experiments were highly reproducible and of sufficient quality to permit robust analysis.

### Global transcriptome analysis identified temperature-regulated genes

**Figure 3:**
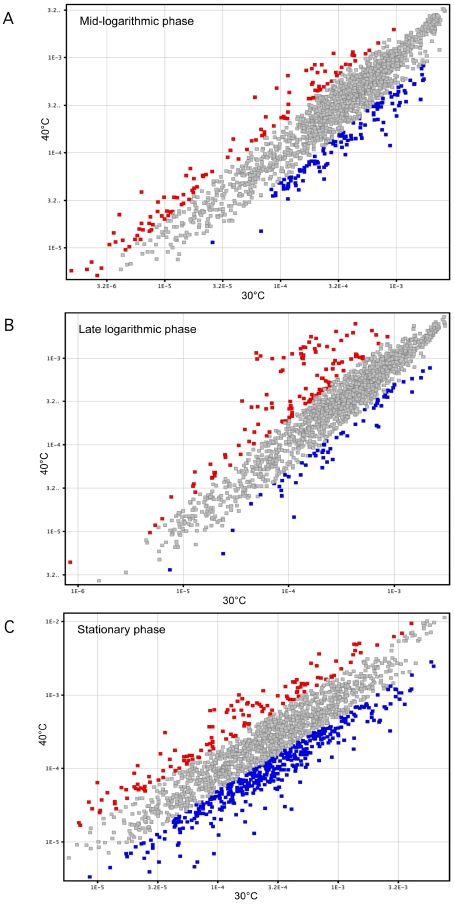
Expression of the 1,995 genes. The scatter diagrams display normalized spot intensities of the microarray analysis from GBS strain NEM316 incubated at 30°C (horizontal axis) and 40°C (vertical axis). Genes significantly up-regulated 2-fold or more at 40°C relative to 30°C are in red, and genes up-regulated 2-fold or more at 30°C relative to 40°C are in blue. Only genes for which a statistically significant difference (*P*<0.05) was observed are indicated for (A) mid-logarithmic phase, (B) late logarithmic phase, and (C) stationary phase.

Results (normalized expression values) obtained for all 1,995 probe sets (corresponding to open reading frames, ORFs) represented on the chip are presented as a supplementary table (Table S1). The data reveal extensive transcriptome differences between bacteria grown at 30°C and 40°C at the same point in their growth curve. Nine to 26% of the genes were differentially expressed at one or more time points studied. The majority of significant transcript changes occurred in the stationary phase of growth (513 differentially expressed genes). In addition, we identified 225 and 177 differentially-expressed genes in the mid- and late logarithmic phases, respectively (Fig. 3). Interestingly, most of the changes in stationary phase were observed in organisms grown at 30°C, whereas changes in late logarithmic phase were observed mostly for bacteria cultured at 40°C (Table 1).

**Table 1:** Number of transcripts significantly up-regulated at 30°C and 40°C as a function of the stage of bacterial growth.

	30°C	40°C
mid-logarithmic phase	120	105
late logarithmic phase	62	115
stationary phase	394	119

**Figure 4:**
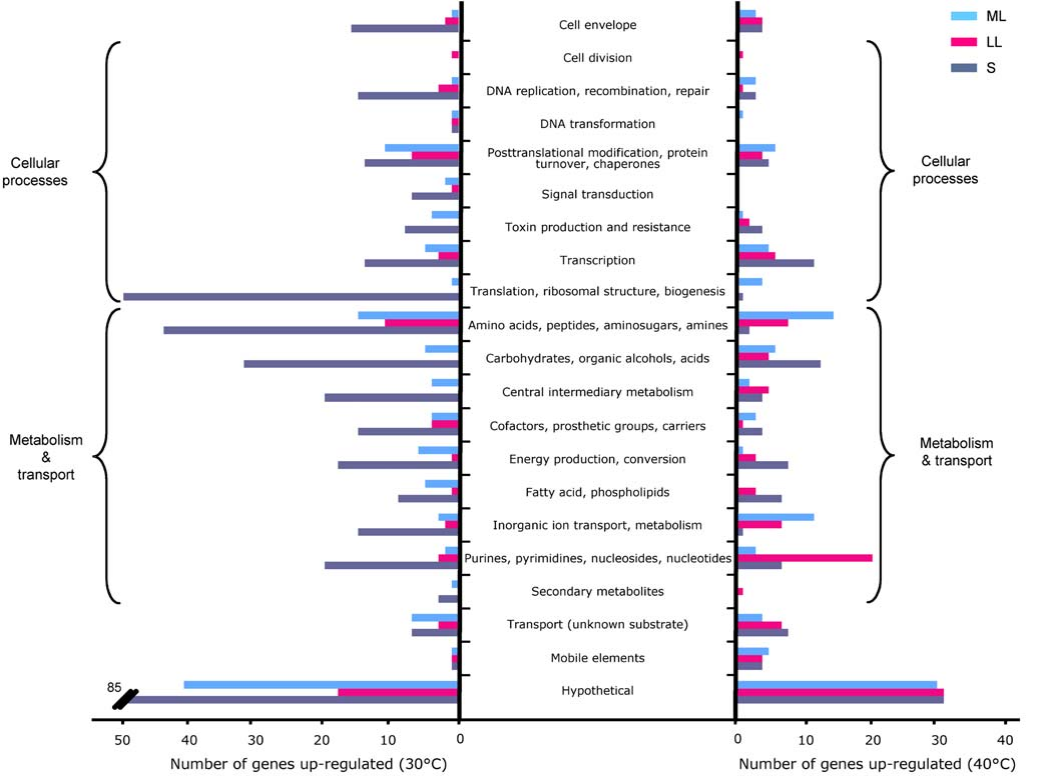
Differential regulation of gene expression in GBS strain NEM316 at 30°C and 40°C. Genes were classified into 21 main functional categories. Bars indicate the numbers of genes up-regulated at one temperature in mid-logarithmic (ML), late logarithmic (LL), and stationary (S) phase.

For ease of analysis and description, the GBS genes were assigned to 21 functional categories based on their involvement in metabolic processes or “cell maintenance” functions (Fig. 4). A prominent finding was that a large proportion of genes encoding proteins involved in purine and pyrimidine metabolism was up-regulated in the late logarithmic phase at 40°C relative to 30°C, but also relative to mid-logarithmic and stationary phases at 40°C (see below) (Fig. 4). It is also notable that more than 10% of the genes encoded by mobile and extrachromosomal elements were up-regulated at 40°C, regardless the phase of growth (Fig. 4). In contrast to the findings noted above for the purine/pyrimidine metabolism genes, many genes involved in metabolism, cellular processes, and cell envelope were up-regulated at 30°C relative to 40°C in stationary phase (Fig. 4).

### Changes of GBS transcriptome in stationary phase

Unexpectedly, we did not observe large differences in growth between tested temperatures, suggesting good adaptation of GBS to various growth temperatures. However, despite almost equal CFU/mL in stationary phase (Fig. 1), we observed large number of differentially-expressed genes (Table 1). About 400 genes were up-regulated at 30°C relative to 40°C. Majority of up-regulated genes belonged to categories involved in bacterial metabolism (183 genes, i.e. 23.8% of the genes of these categories present on the chip) and of most of the categories involved in cellular processes (109 genes, i.e. 24.7% of the genes of these categories present on the chip), except genes involved in cell division and DNA transformation (Fig. 4). Conversely, only 54 and 25 genes involved in general metabolism and cellular processes, respectively, were up-regulated at 40°C in stationary phase. Thus, a larger proportion of genes involved in housekeeping functions were expressed at higher levels at 30°C. It needs to be mentioned that approximately 20% of genes changed in stationary phase of growth are of unknown function. The observed differences in expression of metabolic genes could be explained by changes in medium composition and nutrient exhaustion during bacterial growth. Taking into account both the growth data and the microarrays results, it can be speculated that GBS is able to growth easily in a relative wide-range of temperatures and environments. These results are consistent with a large capacity of adaptation of GBS to its multi-hosts lifestyle. On the contrary exclusive human pathogens as *Streptococcus pneumoniae* show greater variability of growth at different temperatures [14].

### Growth temperature influences expression of transcriptional regulators

Most of the homologues of transcriptional regulators present in the genome of strain NEM316 were expressed at similar levels at 30°C and 40°C. For example, the transcript levels of genes encoding the two-component systems CovS/CovR (gbs1671–gbs1672), Stp1/Stk1 (gbs0306–gbs0307), CiaH/CiaR (gbs1019–gbs1020), and VicR/VicK (gbs0741–gbs0742) were similar at both temperatures, primarily at mid- and/or late logarithmic phases. However, expression of several other regulatory genes was significantly influenced by growth temperature, predominantly genes implicated in carbohydrate metabolism, ion uptake, and cell envelope synthesis. Gbs0191 (encoding a BglG family protein) and gbs0267 (encoding a Mga-like protein), both implicated in sugar metabolism, were 2.3- and 3.1-fold up-regulated, respectively, at 30°C in stationary phase. *adcR* (gbs0150) which encodes the regulator of the AdcCBA high-affinity zinc uptake system was up-regulated 5.8-fold.

Other genes encoding proven and putative transcriptional regulator homologues were up-regulated at 40°C relative to 30°C, including gbs0048 (a putative Cro/CI family regulator), gbs0618 (a putative TetR family regulator), gbs0685 (an uncharacterized DNA-binding response regulator), gbs0857 (a putative TenA family regulator), and gbs0627 (a transcriptional regulator of the AraC family). Although many transcriptional regulators are predominantly involved in bacterial metabolism, recent work has demonstrated that they also play a direct role in virulence. For example, in GAS the catabolite control protein A (CcpA) is a global regulator of carbohydrate utilization genes, and it also directly influences transcription of proven virulence factors [15]. In an analogous way, this strategy may allow GBS to link damage to the host with its overall metabolic status [16], while adapting to new nutrient conditions encountered in various environments [17].

### Stress protein genes are differentially and sequentially expressed at 30°C and 40°C

GBS has homologues of three of the four heat shock response regulatory proteins identified in *Bacillus subtilis* including class I, class III, and class IV heat shock proteins [18]. For most of the genes implicated in GBS stress adaptation, we observed an up-regulation at 30°C relative to in mid-logarithmic and/or late logarithmic phases, whereas the same genes were up-regulated at 40°C relative to 30°C in stationary phase. Transcript changes occurred in the class I heat shock *hrcA*-*grpE* operon (gbs0094–gbs0095) and *clpL* (gbs1376; class III heat shock family). The transcripts levels of three other genes also were similar at 30°C and 40°C during the stationary phase: 1) gbs0756, encoding a stress-responsive transcriptional regulator similar to PspC, 2) gbs1721, belonging to a universal stress protein family, and 3) two genes encoding stress proteins similar to the Gls24 family (gbs1202 and gbs1204). In some cases, genes were only differentially expressed at one temperature. For example, *clpE* and *htr*, and *clpA* and *ftsH* were only upregulated at 30°C, at mid-logarithmic phase or stationary phase, respectively. Similarly, gbs0625 and gbs1982, encoding a chaperone protein and a DNA damage inducible protein respectively, were only up-regulated at 40°C. Another class I operon, *groES*-*groEL*, and genes encoding other chaperone proteins (gbs0422, gbs1383, gbs1634 (*clpX*), gbs1611, gbs1634 (*clpP*), and gbs1865) were transcribed at high levels at both temperatures but there was no significant difference between 30°C and 40°C at any growth point. Such results underscore the great complexity of the bacterial stress response induced by growth temperature, with mechanisms involving proteins always required during bacterial growth, whereas others are only necessary during a specific step of the growth.

To the difference of heat-shock proteins that seem necessary to the bacteria both at 30°C and 40°C, *cspC* (gbs2053) is always 3-fold up-regulated at 30°C. This is consistent with the major role of the cold-shock proteins of the Csp family on transcriptional regulation, post-transcription regulation, and translation control under low temperature [19]. Other genes encoding cold shock-induced proteins, such as the RNA helicase *deaD* (gbs0797), the DNA gyrase *gyrA* (gbs0948), and the polyribonucleotide nucleotidyltransferase *pnp* (gbs0198), are also up-regulated at 30°C in stationary phase. Interestingly, all of these genes (except *gyrA*) encode class I shock proteins, which are induced at high levels after a shift to a lower temperature (*deaD* is more than 8-fold up-regulated at 30°C) whereas class II shock proteins are induced a few-fold from their steady-state levels after a downshift in temperature [20]. Thus, these proteins are not only necessary immediately after the cold shock but also after a longer period of growth at low temperature.

### Expression of proven and putative virulence genes is modified by temperature

Several proven or putative virulence genes were differentially expressed in response to growth temperature changes.

#### (i) Hemolysins

**Figure 5:**
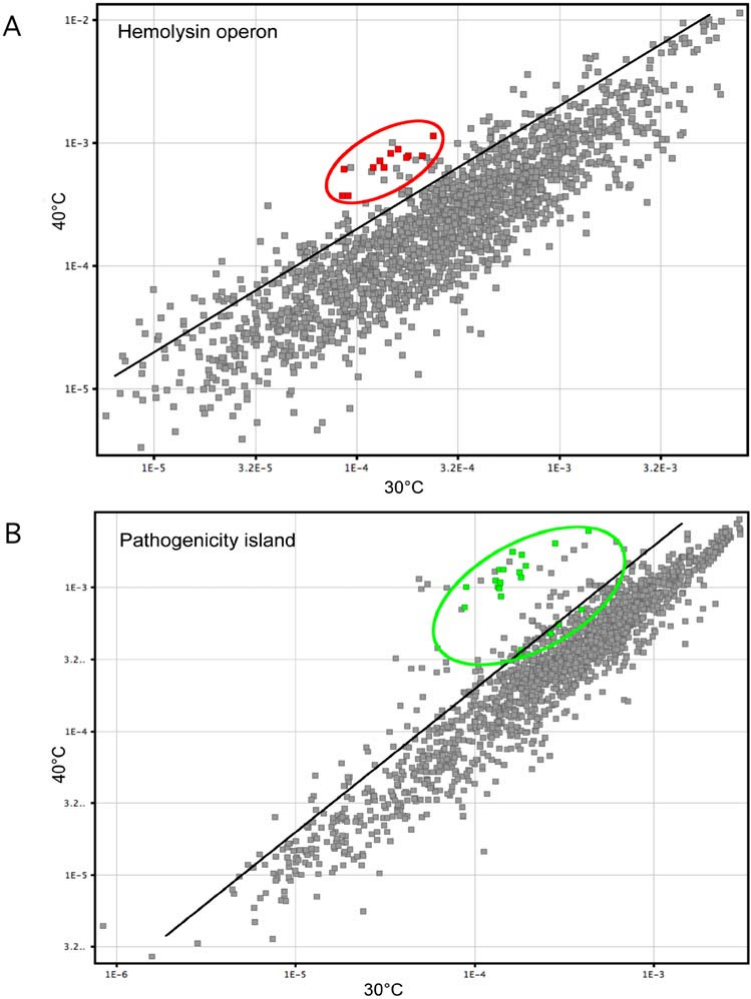
Scatter graph showing the plots representing genes of specific interest. (A) the genes of the hemolysin operon (gbs0644–gbs0655) in red (stationary phase); (B) the genes of putative pathogenicity island IX (gbs1053–gbs1076) in green (late logarithmic phase).

We observed differential expression of gbs0644–gbs0655, a 12-gene cluster required for GBS hemolytic activity [21]. This cluster contains genes encoding the hemolysin molecule (*cylE*) [22], an ABC transporter (*cylA*, *cylB*), an acyl carrier protein homologue (*acpA*), proteins involved in fatty acid biosynthesis (*cylD*, *cylG*, *cylZ*, *cylI*), putative transferases (*cylF* and *cylJ*), and two GBS-specific ORFs of unknown function (*cylX* and *cylK*) [23]. In the aggregrate, genes in this cluster were up-regulated 1.6- to 2.2-fold at 40°C relative to 30°C (6 genes were up-regulated >2-fold) in late logarithmic phase, and notably they were up-regulated 3.7- to 7.2-fold more at 40°C in stationary phase (Fig. 5A). Similarly, the transcripts for *cylE* were up-regulated 1.9- and 4.8-fold at 40°C in late logarithmic and stationary phase, respectively. GBS has three other putative hemolysins [24], and one of them (gbs1389, encoding hemolysin III) also was up-regulated at 40°C (3.3-fold) compared to 30°C. Interestingly, and consistent with our findings with GBS, the transcripts of genes involved in hemolysin synthesis in GAS also were influenced by growth at 40°C [8].

**Figure 6:**
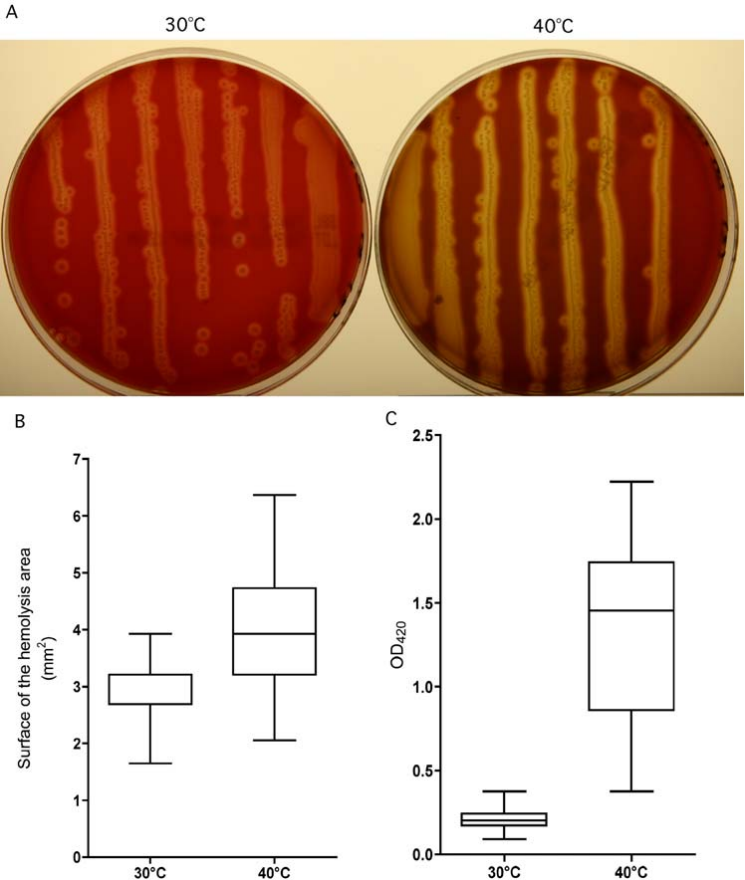
Difference in hemolysis and hemolytic activity of GBS strain NEM316 between incubation at 30°C and 40°C. (A) Hemolysis zones on 5% sheep blood agar plates incubated overnight at 30°C and 40°C. The inoculum used was obtained from a stationary phase THY culture. (B) Surface of the hemolysis area was measured for 100 colonies randomly selected from blood agar plates after incubation as described above. The size of the hemolysis of the colonies after incubation at 40°C is larger than after incubation at 30°C (*P*<0.001). (C) Hemolytic activity measured by turbidimetry (OD_420_) after 1 h incubation at 37°C with 100 µL of the bacterial suspension (10^7^ CFU) and 100 µL of 1% sheep erythrocytes in PBS-glucose. Bacterial suspension from THY broth culture incubated at 40°C has a higher hemolytic activity than bacterial suspension from THY broth culture incubated at 30°C (*P*<0.001). The results are based on assay of 12 independent cultures performed on 3 different days.

To confirm that production of hemolysins increased at high temperature (40°C) in stationary phase, we compared the hemolytic activity of NEM316 grown on blood agar at 30°C and 40°C. Consistent with the transcript data, the zone of hemolysis was significantly smaller at 30°C than at 40°C (Fig. 6a). The average hemolytic area for bacterial colonies grown at 30°C was 2.8 mm^2^, whereas at 40°C the average was 4.2 mm^2^ (*P*<0.001) (Fig. 6b). We also performed quantitative assays by incubating bacterial cells at 30°C and 40°C in THY, and then testing their hemolytic activity at 37°C, to avoid influence of difference in temperature during this last experimental step. We found significantly higher hemolytic activity for bacteria grown at 40°C compared to bacteria grown 30°C (*P*<0.001) (Fig. 6c).

It has been shown previously that the hemolytic activity of GBS increases with temperature [25], [26]. However, here we demonstrated that the hemolysin production itself is also enhanced at high growth temperature. Furthermore, we showed that not only *cylE* but all of the *cyl* operon is up-regulated at 40°C, which is consistent with the fact that most if not all of the genes of the operon are necessary for a complete hemolytic phenotype [23], [27].

#### (ii) DNAse

Two genes with homology to the *spd3* gene encoding streptodornase (DNase) in GAS [28] were up-regulated at 40°C relative to 30°C. gbs0609 was up-regulated in mid- and late logarithmic phase (3.6- and 6-fold, respectively), and gbs0153 was up-regulated in stationary phase (2.3-fold).

#### (iii) Pathogenicity island

We discovered that the gene cluster corresponding to putative pathogenicity island IX (gbs1053–gbs1076) in strain NEM316 was up-regulated at 40°C. Pathogenicity island IX has genes with homology to those encoding a two-component regulatory system, a carbon starvation protein, and several secreted proteins [29]. In contrast to pathogenicity islands that are specific for strain NEM316, island IX was present in five of the other available sequenced genomes [30], suggesting a putative role of this cluster in virulence. The gbs1053–gbs1057 genes were up-regulated ∼2-fold, whereas genes in the gbs1061–gbs1076 region were expressed greater than 5- to 11-fold at 40°C relative to 30°C (Fig. 5B).

#### (iv) Genes encoding putative extracellular proteins (LPXTG/LPXTN motifs)

In contrast to GAS, relatively little is known about the function of putative extracellular proteins made by GBS. Many of these proteins may participate in adherence to host molecules during colonization or invasion. There was no significant difference in transcript levels between 30°C and 40°C for genes involved in the synthesis of the two known pilin systems in GBS. In contrast, several other genes encoding putative cell-surface proteins with a LPXTG/LPXTN cell-wall anchoring motif were differentially expressed according to the temperature. Interestingly, gbs1529 (*srr-1*), encoding a serine-rich repeat protein involved in adherence [31], gbs1308 (*scpB*), encoding a bifunctional protein which cleaves the complement component C5a and mediates adherence to fibronectin [32], and gbs2018 (*bibA*), which has been implicated in resistance to phagocytic killing and GBS survival in human blood [33], were up-regulated at 40°C. ORFs whose functions are less known, such as gbs1929, gbs0428, gbs1288, and gbs1403, also were up-regulated at 40°C. Proteins encoded by these ORFs may be required for adherence during pathogenesis (colonizing the lungs of neonates) and invasion (blood, cerebrospinal fluid), thus providing distinct advantages to the bacterium during the initial steps of infection.

Conversely, other ORFs encoding LPXTG proteins, including gbs0393, gbs0451 (*cspA*, a cell-surface protease which cleaves fibrinogen) [34], gbs2008, and the putative operon gbs1143–gbs1145 were up-regulated at 30°C. Differential expression of genes encoding proteins with LPXTG motifs is consistent with the idea that GBS must adhere to several cell types in the course of its life cycle.

### Transcription of genes encoding iron homeostasis proteins is significantly increased at 40°C

It is well known that differential expression of genes encoding proteins involved in iron homeostasis proteins occurs in response to growth temperature [8], [35]. In this regard, we found that transcription of many GBS genes involved in iron homeostasis was influenced by growth temperature. We found that all genes in the *fhuCDBG* operon (gbs1462–gbs1465) encoding a siderophore-dependent iron transporter [36] were up-regulated at 40°C at mid-logarithmic and/or late logarithmic phases. These findings are analogous to data reported previously for the homologous *ftsABCD* operon in GAS grown at 40°C [8]. The homologue of the GAS *mtsABC* operon (gbs1587–gbs1589) was up-regulated 2- to 2.3-fold at 40°C relative to 30°C. MtsABC is involved in ferric and manganese ion uptake in several low GC% gram-positive bacteria, and plays a role in GAS stress resistance and virulence [37]. A third putative operon (gbs1042–gbs1045) involved in iron uptake and transport in *S. mutans* [38] was also up-regulated 1.7 to 2.1 at 40°C in GBS, as were two other genes, gbs0563 and gbs1112, both encoding iron-sulfur cluster-binding proteins. In addition, we found that genes implicated in nickel metabolism (gbs1573–gbs1577), were also up-regulated at high temperature. Conversely, and consistent with these data, gbs1749 encoding an iron-dependent repressor was down-regulated at 40°C relative to 30°C. The need of iron by bacteria *in vivo* during infection when the amount of free iron is low is well known. Our results indicate that the expression of iron metabolism genes also is increased in GBS in response to elevated temperature, as observed for GAS and *Escherichia coli* [8], [35].

### Transcription of genes involved in purine and pyrimidine metabolism is enhanced at 40°C in late logarithmic phase

**Figure 7:**
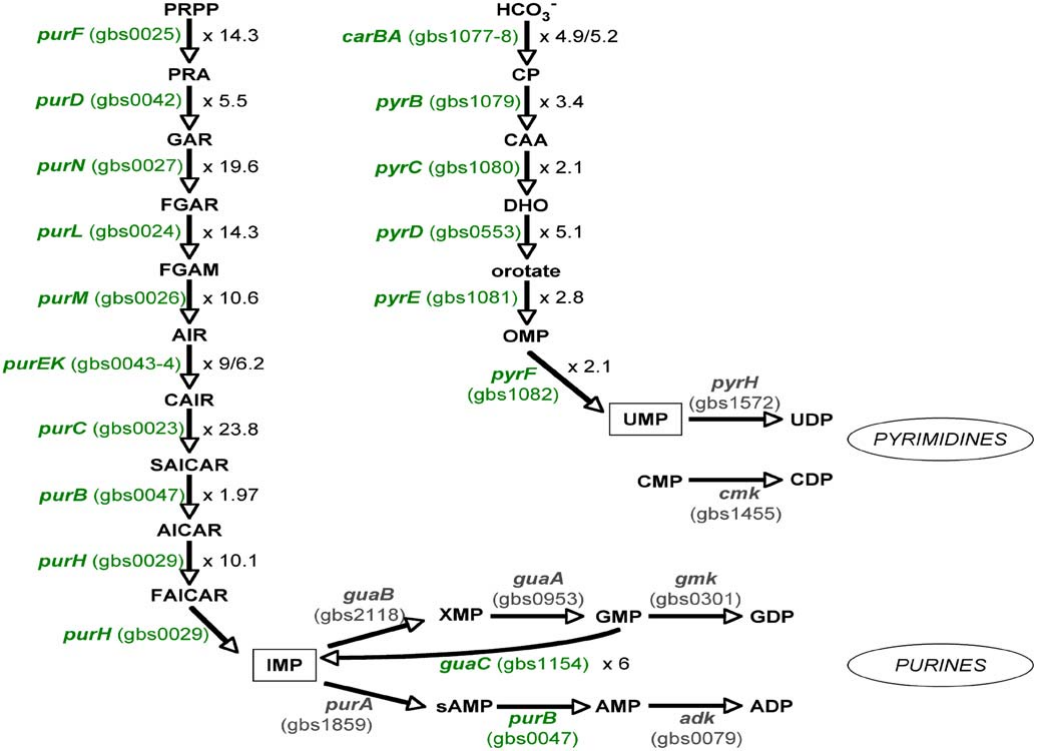
Predicted purine and pyrimidine biosynthetic pathways in GBS. Genes involved in the purine and pyrimidine biosynthetic pathways are up-regulated at 40°C relative to 30°C in late logarithmic phase are in green (level of up-regulation is also indicated). The final products of the enzymes encoded by the up-regulated genes are inosine monophosphate and uridine monophophate (black box). ORFs are named and numbered based on the strain NEM316 genome (Glaser et al., 2002). Enzymes encoded by the genes, *purF*, phosphoribosyl pyrophosphate amidotransferase; *purD*, glycinamide ribonucleotide synthase; *purN*, glycinamide ribonucleotide transformylase; *purL*, formylglycinamidine ribonucleotide synthase; *purM*, aminoimidazole ribonucleotide synthase; *purEK*, phosphoribosyl carboxyaminoimidazole synthase; *purC*, succinocarboxyamide carboxyaminoimidazole ribonucleotide synthase; *purB*, adenylosuccinate lyase; *purH*, bifunctional aminoimidazole carboxamide ribonucleotide transformylase/ inosine monophosphate cyclohydrolase; *purA*, adenylosuccinate synthase; *adk*, adenylate kinase; *guaB*, inosine monophosphate dehydrogenase; *guaA*, guanosine monophosphate synthase; *gmk*, guanylate kinase; *guaC*, guanosine monophosphate reductase; *carAB*, carbamoylphosphate synthase; *pyrB*, aspartate transcarbamoylase; *pyrC*, dihydroorotase; *pyrD*, dihydroorotate dehydrogenase; *pyrE*, orotate phosphoribosyltransferase; *pyrF*, orotate monophosphate decarboxylase; *pyrH*, uridine monophosphate kinase; *cmk*, cytosine monophosphate kinase. Abbreviations for metabolites, PRPP, phosphoribosyl pyrophosphate; PRA, phosphoribosyl amine; GAR, glycinamide ribonucleotide; FGAR, formylglycinamide ribonucleotide; FGAM, formylglycinamidine ribonucleotide; AIR, aminoimidazole ribonucleotide; CAIR, phosphoribosyl carboxyaminoimidazole; SAICAR, succinocarboxyamide carboxyaminoimidazole ribonucleotide; AICAR, aminoimidazole carboxamide ribonucleotide; FAICAR, formaminoimidazole carboxamide ribonucleotide; IMP, inosine monophosphate; XMP, xanthosine monophosphate; GMP, guanosine monophosphate; GDP, guanosine diphosphate; sAMP, adenylsuccinate; AMP, adenosine monophosphate; ADP, adenosine diphosphate; HCO_3_^−^, bicarbonate; CP, carbamoylphosphate; CAA, carbamoylaspartate; DHO, dihydroorotate; OMP, orotate monophosphate; UMP, uridine monophosphate; UDP, uridine diphosphate; CMP, cytosine monophosphate; CDP, cytosine diphosphate.

Many genes involved in nucleotide metabolism were up-regulated in late logarithmic phase at 40°C. For example, ten genes *purC/purL/purF/purM/purN/purH/purD/purE/purK/purB* (gbs0023–gbs0027, gbs0029, gbs0042–gbs0044, gbs0047) involved in purine synthesis were 5- to 24-fold up-regulated. These genes are all implicated in the *de novo* purine biosynthetic pathway responsible for the synthesis of inosine monophosphate (Fig. 7). Interestingly, we found no difference in the expression of genes encoding enzymes of guanosine synthesis, whereas the gene *guaC* (gbs1154), which encodes an enzyme transforming guanosine monophosphate into inosine monophosphate, was transcribed at high levels and up-regulated 6-fold at 40°C. Similarly, *carA/carB*, *pyrB/pyrC/pyrE/pyrF* (gbs1077–gbs0082), and *pyrD* (gbs0553), all involved in pyrimidine biosynthesis, were 2- to 5-fold up-regulated at 40°C (Fig. 7).

Consistent with the findings about purine and pyrimidine metabolism genes, mutations in purine biosynthetic genes attenuate virulence in several bacteria including GBS [39], [40]. Recently, the importance of *de novo* purine and pyrimidine biosynthesis was demonstrated for growth of several pathogens in human serum [41]. Our results now provide evidence for the influence of the temperature of incubation on the expression of these genes.

### Summary

**Figure 8:**
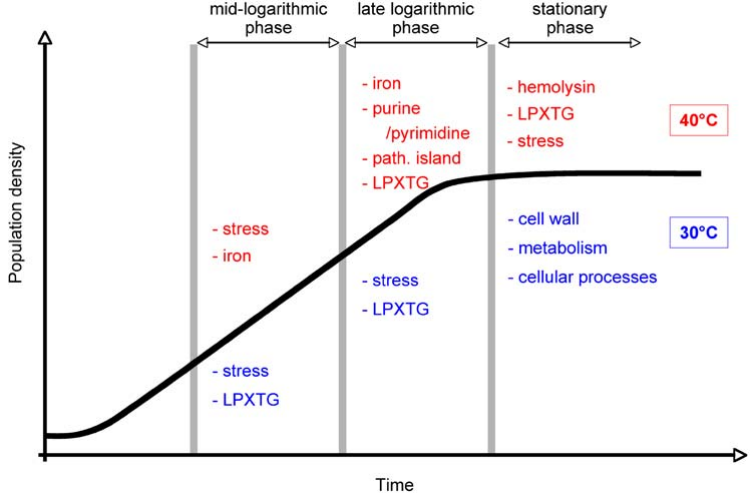
Schematic summarizing the transcriptional response of GBS to change in growth temperature. The temporal order of gene induction is shown for mid-logarithmic, late logarithmic, and stationary phases. Only the main representative changes are presented for one temperature relative to the other. Transcript changes at 40°C are in red and changes at 30°C are in blue.

Our results provide a genome-wide expression profile view of the response of the GBS transcriptome to growth at 30°C and 40°C. Notably, the organism differentially regulates expression of genes involved in stress response, transcriptional regulation, metabolism, and virulence at different stages of growth (Fig. 8). This study documents that temperature is a key signal able to trigger extensive modification in the GBS transcriptome. In the aggregate, the data provide new leads for molecular pathogenesis research, and it would be interesting to confirm the influence of temperature in *in vivo* conditions, especially when variations of temperature between physiological and pathological conditions are of lower magnitude.

## Materials and Methods

### Bacterial growth conditions

Serotype III GBS reference strain NEM316 was used in this study [18]. This strain was isolated from a fatal case of invasive infection and its genome has been sequenced and annotated [18]. Bacteria were cultured in Todd Hewitt broth supplemented with 0.2% yeast extract (THY) in 5% CO_2_ at 37°C overnight without shaking, then used to inoculate (1:50 dilution) triplicate cultures of fresh THY broth equilibrated at 30°C or 40°C. Bacteria were cultured stationary at 5% CO_2_, and growth was evaluated by monitoring OD_600_ and CFU plate counting. Bacterial cells were collected for RNA isolation in mid- and late logarithmic phase, and 3 h after the bacteria entered stationary phase.

### RNA isolation

Each bacterial sample used for RNA isolation was treated with 2 volumes of RNAprotect Bacteria Reagent (Qiagen, Valencia, CA), collected by centrifugation, and stored at −80°C. Bacteria were suspended in 200 µL Bacterial Enhancement Reagent (Invitrogen, Carlsbad, CA) and incubated 4 min at 95°C. RNA was extracted using Lysing Matrix B microtubes containing 0.1 mm silica spheres (Qbiogene, Carlsbad, CA) and 1 mL Trizol (Invitrogen) and a FastPrep FP120 cell disrupter (Qbiogene). RNA extraction was completed with the RNeasy Mini kit (Qiagen) according to the manufacturer's instructions. Samples were treated with DNAFree (Ambion, Austin, TX) to remove trace DNA, and 40 cycles of PCR were performed with RNA templates to ensure the absence of contaminating genomic DNA. RNA concentration was evaluated by measuring absorbance at 260 and 280 nm, and RNA quality was evaluated by electrophoretic analysis with an Agilent 2100 Bioanalyzer (Agilent Technologies Inc., Palo Alto, CA).

### cDNA synthesis, fragmentation and labelling

The methods used for cDNA synthesis, fragmentation, and labelling have been described extensively [42]. Briefly, cDNA synthesis was performed with 4.5 µg of random primers (Invitrogen, Carlsbad, CA) that were added to each RNA sample, annealed 10 min at 70°C and 10 min at 25°C. First-strand cDNA was synthesized with 1,500 U of SuperScript III (Invitrogen) in the presence of 0.5 mM dNTPs, 30 U SUPERaseIn RNase inhibitor (Ambion), and 1 mM dithiothreitol (10 min at 25°C, 60 min at 37°C, 60 min at 42°C, 10 min at 70°C). RNA was removed by alkaline hydrolysis (final concentration of NaOH, 30 min at 65°C), and neutralized with HCl. cDNA purification was performed using the QIAquick 96 kit (Qiagen) according to the manufacturer's recommendations, except that an extra 10-min centrifugation was used to remove traces of phycoerythrin-ethanol buffer. For cDNA fragmentation, 2 µg of cDNA and 1.2 U of DNase I Amp Grade (Invitrogen) were used (10 min at 37°C, then 10 min at 93°C). RNA quality was evaluated by electrophoretic analysis with an Agilent 2100 Bioanalyzer after cDNA synthesis and cDNA fragmentation. The fragmented cDNA was 3′ end-labeled with biotin-ddUTP using the gene chip DNA labelling kit (Affymetrix, Santa Clara, CA) (60 min at 37°C) according to the manufacturer's instructions.

### Expression microarray hybridization and analysis

Expression microarray analysis was performed with a custom-made Affymetrix chip formulated based on the genome sequence of strain NEM316 [18]. The chip contains 1,995 probe sets corresponding to the annotated ORFs in this genome. Briefly, end-labeled cDNA was hybridized overnight at 40°C using the Affymetrix hybridization and staining modules, according to the manufacturer's instructions. Chip hybridization data were acquired and normalized using Affymetrix GeneChip Operating Software (GCOS). Hybridization intensity values were normalized to the mean intensity of all GBS genes present on the chip using GCOS version 1.0 to permit comparison of data obtained from multiple experimental conditions. Only genes with a “present” signal were analyzed further. Data obtained from biological replicates of each experimental condition derived from three independent cultures were used in the analysis. A principal component analysis (PCA) was performed using the Partek Pro 6.0 package (Partek, Saint Louis, MO), and a visualization system was used to assess microarray quality and array-to-array variability. Input information combined hybridization intensity values and information about sample preparation and hybridization. An ORF was considered to be differentially expressed at 30°C or 40°C if there was a significant (*P*<0.05, T-test) change in expression greater than 2-fold at one or more time points (that is, in mid- or late logarithmic phase, or stationary phase). ArrayAssist software v5.5 (Stratagene, La Jolla, CA) was used to perform analyses and generate graphs.

The microarray data of this study have been deposited in the Gene Expression Omnibus database (GSE11666).

### Agar-plate hemolysis assay and hemolytic activity

Strain NEM316 was cultivated in THY in 5% CO_2_ at 30°C and 40°C. Stationary-phase bacteria were grown on 5% sheep blood agar plates and incubated overnight at 30°C and 40°C. Hemolysis was evaluated visually and by measurement of the hemolytic zone with the PDQuest™ 2-D Analysis Software (Bio-Rad Laboratories, Hercules, CA).

Assays for hemolytic activity were performed as previously described [43], with minor modifications. Briefly, bacterial cells grown at 30°C or 40°C in THY until stationary phase were collected by centrifugation at 3,000×g for 10 min. Cells (10^8^ CFU) were washed in phosphate-buffered saline (PBS) and suspended in 1 mL PBS containing 0.2% glucose. One hundred µL of a suspension of 1% sheep erythrocytes in PBS-glucose were added to 100 µL of the bacterial suspension in a 96-well conical-bottom microtiter plate. One hundred µL of 0.1% sodium dodecyl sulfate and 100 µL of PBS-glucose were added to 100 µL of 1% sheep erythrocytes in PBS-glucose as positive and negative controls, respectively. The plates were incubated at 37°C for 60 min, centrifuged at 3,000×g for 10 min, and 100 µL of the supernatant was transferred to a new plate. The quantity of hemoglobin released was assessed by measuring OD_420_.

## Supporting Information

Table S1 Microarray expression data from strain NEM316 during growth in THY at 30°C and 40°C. Ratios greater than 2 and less than 0.5 (P value less than 0.05) are highlighted in blue and purple, respectively. Mid-logarithmic (ML), late logarithmic (LL), and stationary phases (S).
(0.34 MB PDF)
